# Achromatic flat optical components via compensation between structure and material dispersions

**DOI:** 10.1038/srep19885

**Published:** 2016-01-22

**Authors:** Yang Li, Xiong Li, Mingbo Pu, Zeyu Zhao, Xiaoliang Ma, Yanqin Wang, Xiangang Luo

**Affiliations:** 1 State Key Laboratory of Optical Technologies on Nano-Fabrication and Micro-Engineering, Institute of Optics and Electronics, Chinese Academy of Science, P. O. Box 350, Chengdu 610209, China

## Abstract

Chromatism causes great quality degradation of the imaging system, especially for diffraction imaging. The most commonly method to overcome chromatism is refractive/diffractive hybrid optical system which, however, sacrifices the light weight and integration property of diffraction elements. A method through compensation between the structure dispersion and material dispersion is proposed to overcome the chromatism in flat integrated optical components. This method is demonstrated by making use of silver nano-slits waveguides to supply structure dispersion of surface plasmon polaritons (SPP) in metal-insulator-metal (MIM) waveguide to compensate the material dispersion of metal. A broadband deflector and lens are designed to prove the achromatic property of this method. The method demonstrated here may serve as a solution of broadband light manipulation in flat integrated optical systems.

Flat optical components attracted a large amount of researches due to their advantages of being light weight and often low cost. Most of Flat optical components, such as diffraction grating^1^, Fresnel zone plate^1^ and photon sieve^2^, are based on diffractive optical elements (DOEs). However, DOEs suffer from significant chromatic aberrations: the beam deflection angle and focal length increase and decrease with increasing wavelength, respectively. The chromatism is valuable in the areas, such as spectrum measurement and wavelength-division multiplexing (WDM) in communication, but it is also a serious problem in imaging system, which causes degradation of the imaging quality. To solve this problem, the most common method is refractive/diffractive hybrid optical systems to compensate the dispersion in DOEs by the opposite dispersion in refractive lens^3^. However, combined with traditional refractive lens, systems become heavy and complex, which leads to the satisfaction of the flat, light weight and easy integration properties. The chromatism exists not only in traditional flat optics components, but also in metasurfaces^4^,^5^,^6^,^7^,^8^,^9^,^10^,^11^,^12^,^13^,^14^,^15^,^16^,^17^,^18^,^19^,^20^,^21^,^22^,^23^ which attract great interests for its ultra-thin, ultra-light, flat and integratable properties. The broadband property of metasurface has also been investigated and most of these metasurfaces hold reversed chromatism similar to DOEs^4^,^5^,^6^,^7^.

Chromatic aberration is a functional distortion of the optical component at different wavelengths. The reason is the component’s phase mismatch for different wavelengths no matter in traditional refractive and diffractive optical components or novel metasurface. In refractive optics, the phase is accumulated through propagation in which is decided by physical distance and refractive index *n*(*λ*). For normal material dispersion, the refractive index *n*(*λ*) increases with decreasing wavelength, which causes an extra phase to make phase mismatch at different wavelength. For diffractive optics, owing to the efficient light path is short in DOEs, the material dispersion can be ignored. However, the chormatism generated by structure dispersion appears. For instance, the directions of the beams diffracted by grating depend on the spacing of the grating and the wavelength of the light. For the same grating, the diffracted angle is positively related to the wavelength. To achieve the same diffracted direction, the spacing of the grating should increase while wavelength increases. It can also be interpreted as the spacing of the grating is required to match the equal phase diffractive wavefronts at different wavelength. For metasurface, although dispersionless phase generated by metasurface is realized^7^,^8^,^9^, the phases are not matching the required phase diffractive wavefronts at different wavelength. Most recently, Capasso *et al*. proposed a new method using rectangular dielectric resonator for multi-wavelengths to achieve achromatism via dispersive phase compensation^10^. However, the essence of this work is multi-wavelength achromatic and not continuously broadband achromatic.

In this paper, a method to overcome the above issues suffered by previous flat optical components is proposed by utilizing structure dispersion from surface plasmon polaritions (SPP) mode in metal-insulator-metal (MIM) waveguide to compensate material dispersion from metal for achieving a broadband achromatic plasmonic component (APC). The dispersion properties of silver MIM waveguide is proved broadband achromatic in both theory and finite element simulation at the wavelength λ  =  1000–2000 nm. A flat deflector and lens based on MIM waveguides are validated broadband achromatic as theory analysis predicted. The deflector is designed can maintain broadband achromatic directional radiation at arbitrary incident angle. This method opens opportunities to realize lightweight chromatically-corrected imaging systems and achromatic flat integrated optical systems.

## Results

### Principle and unit cell

To realize any desired functionality (focusing, beam deflection, etc.), the phase retardation of optical component is required to compensate the phase retardation of propagation in free space. For example, in traditional refractive lens, the light path in lens is used to compensate the light path in free space propagation from lens to focus spot to achieve that total light path is a constant. The dispersion of required phase 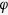 at the point *r* can be generally given by^10^:


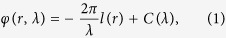


where 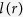 is the physical distance between the interface at position *r* and the desired wavefront. The free parameter 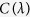 can be set as an arbitrary value to optimize the elements for linear optics applications. Therefore, relative phase distribution 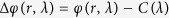 can be used to substitute absolute phase distribution 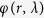. For broadband metasurface^4^,^8^, 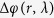 usually does not change at varied wavelength so that the desired 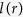
becomes 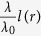 with incident wavelength *λ* when the phase distribution is designed for *λ*_*0*_. The effect of this change is that the beam deflection angle and the focal length are directly and inversely proportional to the wavelength for the functional components, respectively. For an achromatic component, 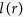, which carry the information for the functionality, should be a constant at the same point *r* so that the desired phase at an arbitrary point *r* is only a function of wavelength so that Eq. (1) can be written as,


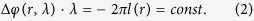


Therefore, there is an assist parameter 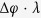 to design the achromatic components. For an achromatic flat component, 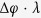 is dispersionless at an arbitrary point.

In recent years, metallic optical waveguides have been of particular interest in plasmonics research due to the evanescent wave magnification and field localization properties of surface plasmon^22^,^23^,^24^,^25^,^26^,^27^,^28^,^29^,^30^,^31^,^32^,^33^,^34^. The amazing properties of these waveguides are potential to overcome the diffraction limit in conventional dielectric waveguides and to realize nano-scale photonic components for high integration^23^. However, the chromatic aberration is a great challenge in the optical components based on metallic waveguide. For instance, the deflection angle increases obviously with increasing wavelength^23^; the focus points shift distinctly at different wavelength^24^. The chromatism results from the material dispersion and structure dispersion of MIM waveguide. Here we show that these two kinds of dispersion are opposite and can be designed to compensate each other in special conditions.

When a MIM slit with subwavelength width is illuminated by TM polarized radiation, only the SPP mode exists and can be considered as the fundamental mode which produces phase retardation. The complex propagation constant *β* of SPP mode in MIM waveguide is given by the eigenvalue equation^23^:


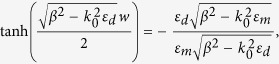


where *w* is the width of slit; *k*_*0*_ is the wave vector of light in free space; *ε*_*d*_ and *ε*_*m*_ are the permittivities of the dielectric medium filled in the slit and the metal, respectively.

The material dispersive behavior of metal 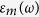 can be estimated by the Drude model^35^:


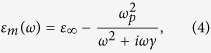


where 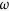 is the angular frequency of the incident electromagnetic radiation, 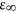 is the permittivity at infinite angular frequency, 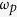 is the bulk plasma frequency which represents the natural frequency of the oscillations of free conduction electrons, and 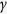 is the collision frequency. At the frequency 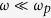 and 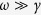, 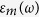 can be approximated to:


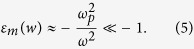


Applying Eq. (5) to simplify Eq. (3), the *βλ* can be written as a function of permittivity of dielectric *ε*_*d*_ and slit width *w*:


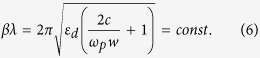


**Figure 1:**
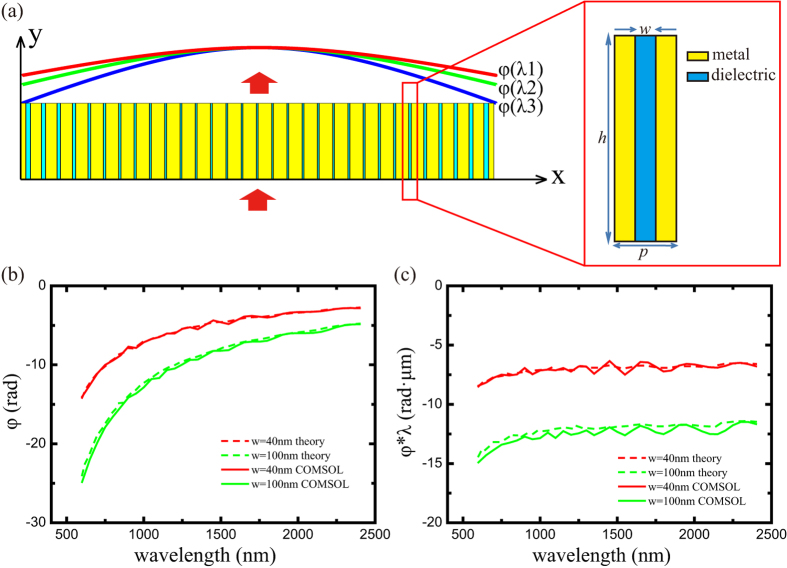
Structural geometry and phase shift dispersion. (**a**) Side view of the achromatic component designed for achromatic lens. The component includes 51 unit cells as shown in the inset. The parameters *w*, *h* and *p* represent the width of dielectric, length of the waveguide and period of the MIM unit cell, respectively. (**b**) Relative phase shift 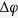 through MIM structure at different wavelength at varied slit width in theoretical calculation and numerical simulation. (**c**) Theoretical and numerical calculated 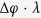 at different wavelength at different slit width.

When the SPP wave passes through the subwavelength metallic slit, the output phase retardation 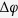 of light transmitted through each slit can be expressed by^23^:


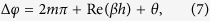


where 
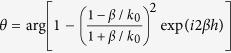
 originates from multiple reflections between the entrance and exit surface^23^; *h* presents the length of the MIM waveguide. Both physical analysis and numerical simulations show that *βh* plays a dominating role in phase shift^23^. Therefore, if we choose *m* = 0, 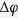 can be approximated as Re(*βh*). The imaginary part of propagation constant of the SPP in the MIM slit is usually ignorable (Im(*β*) ≪1) at the frequency *ω* much higher than collision frequency *γ*. The waveguide length *h* is a constant in components based on MIM slits as shown in Fig. 1(a), so:


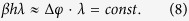


Thus, the MIM waveguide is theoretically proved achromatic at the designed filled dielectric, slit width and slit depth in the frequency range 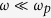 and 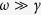.

Furthermore, the dispersive behavior of MIM waveguide is demonstrated in theoretical calculation and numerical simulation. The basic unit of the APC based on MIM waveguide is shown by the schematic cross-section in the inset of Fig. 1(a). The metallic slit width *w* is varied from 20 nm to 100 nm, and the length of waveguide *h* is fixed at 3 μm. The relative permittivity of the material filled in the slit is assumed to be *ε*_*d*_ =  1 for air. Silver is chosen as the metal in this model due to its lower loss.

The dispersion of the MIM silts is shown as Fig. 1(b). 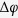 is defined as phase shift compared with the *φ* at slit width *w* = 20 nm. The propagation constant *β* gets the maximum when slit width *w* gets the minimum as Eq. (6), so that 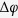 is negative at *w *> 20 nm. A clear trend of the phase shift 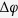 increasing can be seen when decreases slit width *w* or increases wavelength *λ*. Figure 1(c) shows the theoretically calculated and numerically simulated 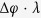 with different silt widths at different wavelengths. 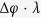 increases rapidly for the increased wavelength below 800 nm and becomes stable at longer wavelength. This phenomenon matches the theoretical analysis that the achromatic effect in our model happens at the frequency 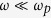 and 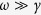. The slight phase oscillation can be explained by Fabry-Perot effect (which is considered as *θ* in Eq. (3)).

### Achromatic light deflection

Light deflector is a basic optical component to deflect the light, which can be realized by optical wedge in refractive optics and grating in diffractive optics. In order to deflect the beam into a spatial orientation with an angle *θ*_0_, the phase retardation 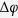 of light transmitted through the slits along the *x* direction should take the form:


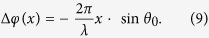


**Figure 2:**
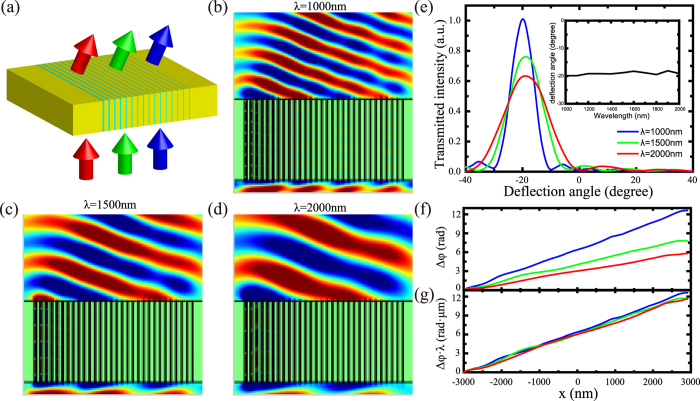
Achromatic plasmonic deflector. (**a**) Schematic configuration of flat achromatic beam deflector. (**b–d**) *Ex* distribution of the deflector at the wavelength λ = 1000 nm, 1500 nm and 2000 nm, respectively. (**e**) Simulated far field intensity as a function of the deflection angle *θ* with normal illumination. Inset: Simulated deflection angle *θ* as a function of wavelength. (**e–f**) Simulated phase shift (**f**) and 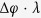 (**g**) at 200nm above the output surface at the wavelength λ = 1000 nm (blue), 1500nm (green) and 2000nm (red).

Based on the aforementioned analysis, an achromatic deflector can be designed by directly arranging the slit width distribution to generate phase shift to match the phase distribution of single wavelength in achromatic range as shown in Fig. 2(a). For instance, a deflector is designed by 29 metallic slits with varies width for deflecting normally incident light to an angle = −19° for λ = 1 μm. The *Ex* distribution of this component with three different wavelengths are shown as Fig. 2(b–d). The beams at three wavelengths are deflected into the same direction and the deflection angles are almost the same as Fig. 2(e). As shown in the inset of Fig. 2(e), the deflection angles are near −19° with deviation less than 5% at the whole wavelength range.

To unveil the essence of the achromatic performance, the phase distributions are analyzed. The simulated phase distributions at different wavelengths are almost linear functions for the spatial coordinate *x* as shown in Fig. 2(f). This result proves that normal incident plane waves are deflected and show expected phase distribution as Eq. (10). As previously demonstrated, 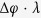 should be a constant at the same position at different wavelengths for an achromatic component. Figure 2(g) shows the calculated 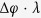 profiles at the output plane of the deflector at different wavelengths, which coincide very well as predicted.

**Figure 3:**
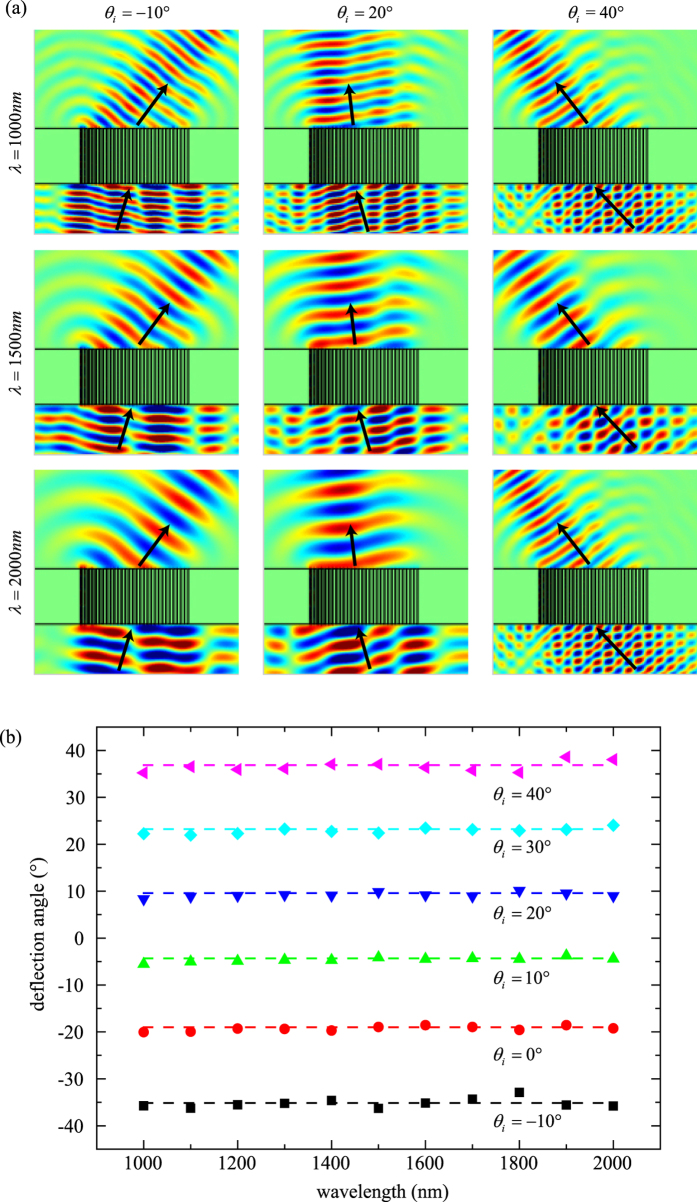
Achromatic properties of deflector with off-axis incidence. (**a**) *Ex* distribution of flat achromatic deflector illuminated by light at the wavelength *λ* = 1000 nm, *λ* = 1500 nm and *λ* = 2000 nm with incident angles *θ*_*i*_  = −10, 20 and 40 degrees. (**b**) Transmitted deflection angles as a function of wavelength at the condition of different incident angle *θ*_*i*_ = −10, 0, 10, 20, 30 and 40 degrees by numerical simulation (points) and theoretical calculation (dashed lines).

Not limited by normal incidence, this achromatic deflector can work when the incident beam off-axis illuminates the component. Figure 3(a) shows the *Ex* distributions at different incident angles (−10°, 20° and 40°) and wavelengths (λ = 1000 nm, 1500 nm and 2000 nm). With the same incident angle, the lights at different wavelengths are deflected into almost the same direction. In theory, the deflection angle is calculated by^14^,^15^:


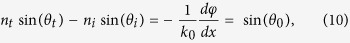


where *θ*_*i*_ is the incident angle, *θ*_*t*_ and *θ*_0_ are the deflection angle with off-axis and normal incidence, respectively. The refractive index *n*_*i*_ is 1.44 for glass substrate and *n*_*t*_ is 1 for the air. Because *θ*_*0*_ is proved achromatic, the deflection angle *θ*_*t*_ can be predicted achromatic at arbitrary incident angle in theory. As shown in Fig. 3(b), the dashed lines are the theoretical deflection angles with different incident angels calculated by Eq. (10), which is unchanged at different wavelengths. The simulated defection angles (points) perfectly match the theoretical defection angles (dashed lines) in a broadband frequency range and at arbitrary incident angles from −10° to 40°, while the deflection angle is from −35° to 37°. Theoretically, the achromatic performance will not change at arbitrary incident angle if the aperture is large enough.

### Achromatic light focusing

The design for a flat achromatic lens based on metallic slits is also presented. The required phase retardation as a function of spatial distance *x* can be calculated as:


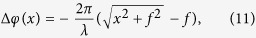


where *f* is the focal length of the plasmonic lens.

**Figure 4:**
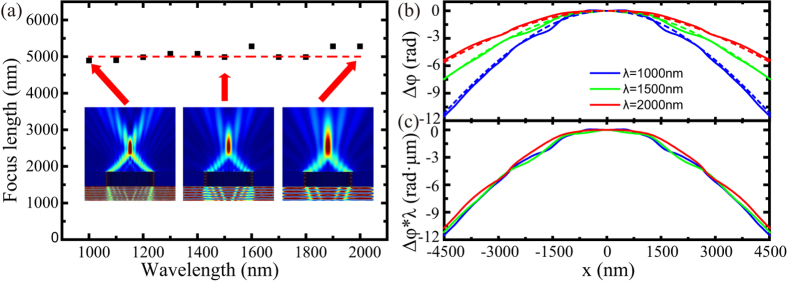
Achromatic plasmonic lens. (**a**) Focus lengths of the designed achromatic lens at different wavelength. Electric field intensity 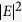 distributions at the wavelength *λ* = 1000 nm, 1500 nm and 2000 nm are shown in the inset. (**b**) Numerical (solid line) simulated and ideal (dashed line) phase distribution at 200 nm above the output surface at the wavelength *λ* = 1000 nm (blue), 1500 nm (green) and 2000 nm (red). (**c**) Simulated spatial distribution of 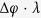 at the wavelength *λ* = 1000 nm (blue), 1500 nm (green) and 2000 nm (red).

The phase distribution is designed for *f* = 5 μm. The number of slits in our design is 51 and the period of the structure is chosen as 200 nm. As shown in Fig. 4(a), the focus length is very closed to 5 μm at different wavelengths. Insets of Fig. 4(a) show the electric field intensity distribution of plasmonic lens illuminated by the light at wavelength of 1000 nm, 1500 nm and 2000 nm, respectively. Although the sizes of focus spot are different, which is determined by diffraction limit, the focus length are the same so this flat lens is achromatic.

To analyze the achromatic performance, the simulated phase distributions at 200 nm above the output plane of the flat lens are shown as solid lines in Fig. 4(b). The dashed lines in Fig. 4(b) stand for the theoretical achromatic distributions for target focus length at different wavelengths obtained from Eq. (9). The simulated phase distributions of output light with different wavelengths show good agreement with the theoretical prediction. Figure 4(c) shows the normalized 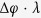 as a function of the *x*-position. Simulated 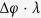 is almost the same, which leads to the same focal length. The slight focus shift can be explained by the little deviation of 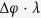.

## Discussion

Compared with the metasurface based on rectangular dielectric resonator^10^ which can get achromatism at only a few discrete wavelengths and encounter much greater deviation at the other wavelengths, our design can hold achromatic performance in such a continuously broadband range *λ* = 1000–2000 nm. Furthermore, this kind of achromatic flat components can avoid complex parameters scanning as the method proposed by Capasso *et al*. Although our design has a challenge in fabrication, it can be realized by the process as shown in Fig. S1.

In addition, theoretically, the achromatic deflection of the MIM based deflector is preserved in arbitrary incident angle, because the MIM waveguide can maintain the desired phase shift by the unique SPP mode at arbitrary incident angle, revealing obvious superiority compared with the achromatic dielectric metasurface with ±1° incident angle tolerance^10^. Actually, aperture is another factor influencing the deflection angle. When the equivalent aperture is as small as wavelength with the increasing incident angle, diffraction affects the deflective behavior.

In summary, we report a method of designing achromatic plasmonic components by compensation between structure dispersion and material dispersion. To demonstrate this method, MIM waveguide is chosen to support structure dispersion of SPP mode and material dispersion of metal. From the theory analysis and simulation results, MIM waveguides can be used to realize achromatic flat component at frequency 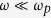 and 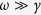. Achromatic deflector and lens are both designed based on sliver slits with variant width at broadband range *λ* = 1000–2000 nm. It is noteworthy that the deflector can maintain a broadband achromatic performance with arbitrary incident angles. Our method opens great potential applications in multicolor stereo imaging and broadband light collection by flat optics components.

## Methods

### Simulation

Numerical simulation results are calculated by COMSOL4.3. A perfect matched layer (PML) as an absorbing boundary condition is used to dissipate outgoing waves. MIM waveguides array are simulated with period of 200 nm. All the simulated phase distributions are at the plane 200 nm above the output surface to minimize the near field noise. The dispersive permittivity of silver is calculated by Drude model^25^ (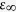 = 4.2; 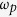 = 1.3 × 10^16 ^rad/s, and 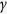 = 9.1 × 10^13 ^rad/s).

## Additional Information

**How to cite this article**: Li, Y. *et al*. Achromatic flat optical components via compensation between structure and material dispersions. *Sci. Rep.* **6**, 19885; doi: 10.1038/srep19885 (2016).

## Supplementary Material

Supplementary Information
