# Supplementary material for: Achromatic flat optical components via compensation between structure and material dispersions
Source: Sci Rep. 2016 Jan 22;6:19885. doi: 10.1038/srep19885 (PMC4726289; doi:10.1038/srep19885)
Supplement: Supplementary Information [file srep19885-s1.doc]

**Supplementary Information**

**Achromatic flat optical components via compensation between structure and material dispersions**

Yang Li, Xiong Li, Mingbo Pu, Zeyu Zhao, Xiaoliang Ma, Yanqin Wang and Xiangang Luo*


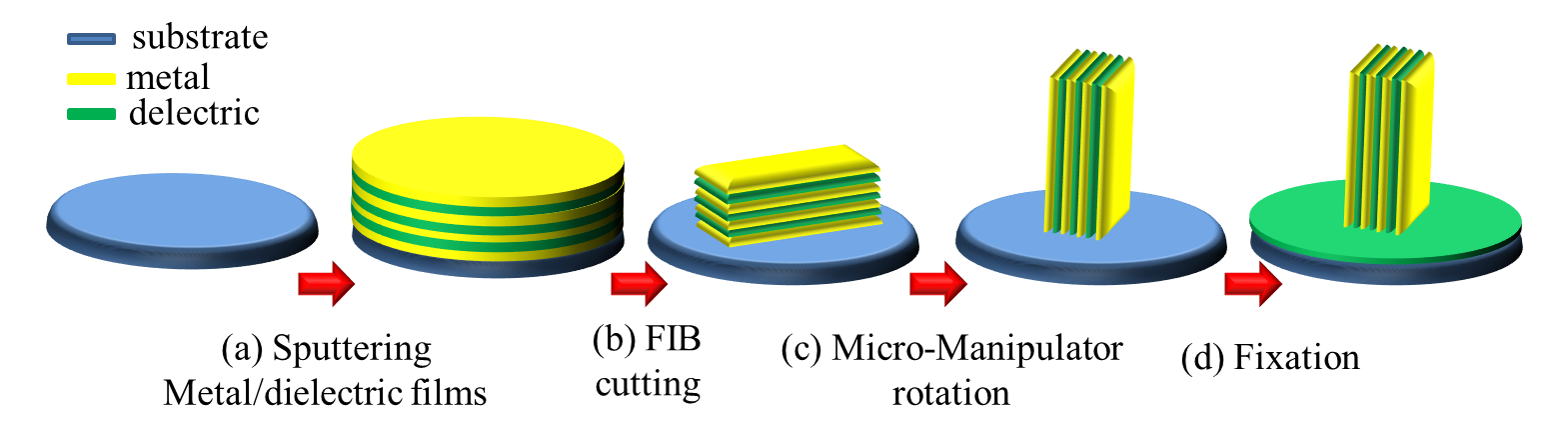


**Supplementary Figure S1. Fabrication process for the design.** (a) Step 1: coating multi-layers film with metal (such as Ag) and dielectric (such as SiO2) on substrate; (b) step 2: cutting the film with 3 μm width and more than tens of micron length by FIB; (c) step 3: moving the multi-layer micro-cube by the micro-manipulator in FIB [1] to another sample or place with a rotation; (d) step 4: fixing the sample by coating a thin film transparent material.

Supplementary reference:

[1] Chai, G., Chow, L., Zhou, D., & Byahut, S. R. Focused-ion-beam assisted fabrication of individual multiwall carbon nanotube field emitter. Carbon **43**, 2083-2087(2005).
